# Identification of a candidate sex determination region and sex-specific molecular markers based on whole-genome re‑sequencing in the sea star *Asterias amurensis*

**DOI:** 10.1093/dnares/dsaf003

**Published:** 2025-01-10

**Authors:** Yanlin Wang, Hongliang Yang, Yixin Wang, Yulong Li, Gang Ni, Scott F Cummins, Muyan Chen

**Affiliations:** The Key Laboratory of Mariculture, Ministry of Education, Ocean University of China, Qingdao, 266003, China; The Key Laboratory of Mariculture, Ministry of Education, Ocean University of China, Qingdao, 266003, China; The Key Laboratory of Mariculture, Ministry of Education, Ocean University of China, Qingdao, 266003, China; CAS Key Laboratory of Marine Ecology and Environmental Sciences, Institute of Oceanology, Chinese Academy of Sciences, Qingdao, 266071, China; The Key Laboratory of Mariculture, Ministry of Education, Ocean University of China, Qingdao, 266003, China; Centre for Bioinnovation, University of the Sunshine Coast, Maroochydore, QLD 4558, Australia; School of Science, Technology and Engineering, University of the Sunshine Coast, Maroochydore, QLD 4558, Australia; The Key Laboratory of Mariculture, Ministry of Education, Ocean University of China, Qingdao, 266003, China

**Keywords:** starfish, whole-genome re-sequencing, GWAS, sex determination, sex-specific markers

## Abstract

Sex determination systems are diverse in echinoderms, however, our understanding is still very limited in this research field, especially for Asteroidea species. The northern Pacific seastar, *Asterias amurensis*, has attracted widespread concern due to its population outbreaks and high-risk invasions. Using whole-genome re-sequencing data from 40 females and 40 males, we identified a candidate sex determination region in *A. amurensis*. Based on the distribution characteristics of 525 sex-associated single nucleotide polymorphisms, identified by GWAS analysis, 119 sex-specific loci were isolated combining a custom Perl script, PCA analysis, and the selection signatures of fixation index F_ST_, suggesting that a 7-12 Mb region on chromosome 10 is a candidate sex-determining region. The existence of female-specific sequences and the genotypes of sex-specific loci indicated that *A. amurensis* might utilize a ZZ/ZW sex-determination system. We also developed two pairs of sex-specific primers that could distinguish the genetic sex of this starfish with 100% accuracy. As the first study on sex determination in Asteroidea, it will provide novel insights into diverse sex determination systems in echinoderms and allow for in-depth studies on sex-related eco-physiological issues in *A. amurensis*.

## 1. Introduction

The northern Pacific seastar *Asterias amurensis* is a keystone predatory starfish that has attracted widespread concern due to its population outbreaks and high-risk invasions. As a benthic echinoderm with a high ability for regeneration and reproduction,^1,2^ it has been reported to pose a serious economic threat to fisheries and aquaculture, as well as disrupt the natural ecological equilibrium.^3,4^ As a successful invasive species, *A. amurensis* is now well-established in Australia because of its high environmental tolerance and long-lived and highly dispersive larvae.^2,5,6^ The Australian government has made considerable efforts for the management and control of this marine pest, including the application of reproductive inhibitors or stimulants.^7–9^ However, the mechanism of its aggregation remains unknown and it is still unclear whether sex pheromones are involved in this process.

Understanding the evolution and maintenance of sexual reproduction, known as the ‘queen of evolutionary biology’, is a fascinating area in the study of life sciences, dictating numerous aspects of an organism’s life history.^10,11^ According to the major factors, sex determination mechanisms can be classified into three categories: genetic sex determination (GSD), environmental sex determination (ESD), and transition of both.^12–14^ Thus far, in marine animals, the most comprehensive studies into sex determination mechanisms have focused on teleost fishes. They possess an enormous variety of approaches that regulate sex determination, ranging from ESD (e.g. water temperature, dissolved oxygen, social status, behavioral control, etc.) to GSD (e.g. ♀XX/♂XY, ♀ZW/♂ZZ, ♀XX/♂XY_1_Y2, ♀XX/♂XO, ♀X_1_X_2_X_1_X_2_/♂X_1_X_2_Y, etc.).^15–17^ Marine invertebrates also encompass various forms of sex determination systems.^18^ They may be quite complex and even differ between closely related species.^19^

Echinoderms are a major group of deuterostome invertebrates with distinct evolutionary classification.^20^ These group of invertebrates possess diverse reproduction modes (hermaphroditism, dioecy, parthenogenesis, and asexual multiplication) and, therefore, serve as excellent subjects for sex determination and sexual plasticity studies.^18,21^ Accumulating studies suggest that their mechanisms of sex determination seem to be diverse. For example, the sea cucumbers (*Apostichopus japonicus*^21,22^ and *Stichopus monotuberculatus*^23^) were reported to have an XX/XY sex-determination system, while the sea urchins *Mesocentrotus nudus*^24,25^ and *Paracentrotus lividus*^26^ may own the ZZ/ZW and XX/XY sex-determination system, respectively. So far, there is no study has been reported on the mechanism of sex determination in Asteroidea, which is known as the most diverse class within the living Echinodermata. Our recent publication reporting a chromosome-level reference genome for *Asterias amurensis* provides a valuable genetic resource for this study.^27^

With the development of rapid and cost-effective next-generation sequencing (NGS) technology, genome-wide association studies (GWAS) via whole-genome re-sequencing has become increasingly popular in studies related to the elucidation of molecular mechanisms for sex determination, including the identification of a sex determination locus and sex-determining genes. This approach has now been widely applied to many aquatic animals, such as the pufferfish *Takifugu bimaculatus*,^28^ the leopard coralgrouper *Plectropomus leopardus*,^29^ the Asian arowana *Scleropages formosus*,^30^ the large yellow croaker *Larimichthys crocea*,^31^ the sea cucumber *A. japonicus*,^21^ the sea urchin *M. nudus*,^24^ and the Pacific abalone, *Haliotis discus hannai* Ino.^32^ Sex-specific molecular markers are also considered to be of great importance for the study on sex-determining mechanisms, particularly for species in which the sex is not morphologically distinguishable,^33^ such as *A. amurensis*. To date, several sex-specific molecular markers have been successfully developed in echinoderms, including the sea cucumbers *A. japonicus*,^22^*S. monotuberculatu*^23^ and *Holothuria scabra*,^34^ and the sea urchin *M. nudus*.^25^ Based on the sex-specific sequences assembled and identified from the whole-genome re-sequencing data, the genetic sex can be distinguished using sex-specific primers coupled with PCR amplification, which is a rapid and reliable method supporting high-throughput operation. In recent years, the NGS method for identifying sex-specific markers has become increasingly popular with the rapid advancement of re-sequencing technology.^23,35–38^

In this study, we identified a candidate sex determination region and sex-specific molecular markers in *A. amurensis* using the whole-genome re-sequencing data from 40 females and 40 males. This provides novel insights into echinoderm sex determination and has implications for exploring the mechanism of *A. amurensis* population outbreaks.

## 2. Materials and methods

### 2.1. Sample preparation

A total of 100 adult *A. amurensis* (average radius of 10.0 cm) were collected by divers in the Qingdao coast area, Shandong Province (China) in December 2022. The sex was identified by gonadal observation with light microscopy. Meanwhile, the gonad and podia tissue from 40 females and 40 males were collected and washed with phosphate-buffered saline (PBS, 1×). These samples were immediately frozen in liquid nitrogen and transferred to −80°C for subsequent experiments. High-quality genomic DNA (gDNA) was extracted from the gonad using DNeasy Blood & Tissue Kit (Qiagen, Germany) according to the manufacturer’s instructions. In addition, 30 adult *A. amurensis* were collected in November 2023, and then gDNA was similarly isolated from podia tissue for subsequent validation of sex-specific markers. All methods for animal experiments were carried out in accordance with the ARRIVE guidelines (https://arriveguidelines.org).

### 2.2. Whole-genome re‑sequencing and genotyping

The quality of gDNA was evaluated using a Nanodrop 2000 spectrophotometer (Thermo Fisher Scientific, USA) and 1% agarose gel electrophoresis. High quality gDNA was fragmented into approximately 350 bp through sonication for whole-genome re‑sequencing library construction. A total of 80 libraries were constructed and sequenced using DNBSEQ-T7 platform to generate 150 bp paired-end (PE150) reads following the manufacturer’s instructions (BGI, China). The clean data was obtained by filtering out low-quality reads using fastp (v0.23.4)^39^ with default parameters.

The clean reads generated by re‑sequencing were mapped to the *A. amurensis* male reference genome^27^ with a size of 491.53 Mb using BWA-MEM2 (v2.2.1),^40^ then samtools (v1.18)^41^ was utilized to sort the output BAM files and remove the PCR duplicates. The Genome Analysis ToolKit (GATK, v4.0)^42^ was used to call single-nucleotide polymorphisms (SNPs). In brief, the HaplotypeCaller algorithm was utilized to call variants individually on each sample and CombineGVCFs was used to merge them. Then, joint genotyping and SNP selection were performed by GenotypeGVCFs and SelectVariants, respectively. SNPs were filtered using vcftools (v0.1.16)^43^ with the parameters ‘--maf 0.05 --min-meanDP 6 --max-meanDP 300 --minGQ 20 --max-missing 0.9 --min-alleles 2 --max-alleles 2’. Genotype-imputation was conducted using Beagle (v5.4).^44^

### 2.3. Genome-wide association study

The GWAS of sex was carried out using plink (v1.90b6.21)^45^ with the parameters ‘--assoc --noweb --allow-no-sex’. The sex phenotype was encoded as 1 (female) and 2 (male). In this study, the significant sex-associated SNPs were selected based on the defined threshold of *P *< 1e−8. The Manhattan plot and QQ-plot were subsequently drawn by CMplot (v4.5.1)^46^ in R. To identify potential sex-determining genes, SnpEff (v4.3t)^47^ was utilized to annotate the significant sex-associated SNPs.

### 2.4. Identification of candidate sex‑determining region

The candidate sex‑determining region was screened based on the number of significant sex-associated SNPs in the Manhattan plot. We defined that the candidate sex‑determination region should be the genome region in which the SNPs are heterozygotic (a minimum of 25 heterozygotes in this study) in one sex and homozygotic in the corresponding sex. Thus, the sex-associated SNPs were examined using the Perl script (https://github.com/lyl8086/find_sex_loci)^48^ with the parameter ‘-c 25 -e 0’ to isolate sex-specific loci. To further demonstrate the accuracy of the sex‑determining region, PCA analysis was conducted using Genome-wide Complex Trait Analysis (GCTA, v1.94.1)^49^ for SNP sets on different chromosomes and genome-wide SNPs. In addition, vcftools (v0.1.16)^43^ was used to calculate fixation index F_ST_ to quantify the allele frequency differences between the two sexes. The genomic region with high F_ST_ values (> 0.25) indicated strong selective signatures.^50^

### 2.5. Screening of candidate sex-specific markers

We randomly selected the clean reads from one female (F04) and one male (M03) *A. amurensis* for genome assembly using SOAPdenovo2 (v2.04) based on the overlapping and pair-end relationship.^51^ After improving the completeness with GapCloser (v1.12),^52^ the scaffold sequences of the two sexes were used as reference genomes for further analysis.

The BWA (v0.7.17-r1188)^53^ was employed to align the sequencing reads of 5 female and 5 male samples to the reference genomes of 2 sexes. Then, the clean reads with two-terminal correct alignments were identified with python for screening of female/male-specific sequences. Based on the re‑sequencing data of 5 females and 5 males, we performed the first round of screening for candidate sex-specific markers. In this study, we focused on the female-specific sequences which could be simultaneously covered by sequencing reads from all female samples but not any male samples. To remove the false-positive sequences, we performed a second round of screening using the pooled data from 30 males and manually removed the invalid sequences. The coverage of candidate female-specific sequences in these samples was further visualized using the Integrative Genomic Viewer (IGV, v2.17.3).^54^

For the female-specific sequences, specific primers were designed using Primer3 (v4.1.0, https://bioinfo.ut.ee/primer3-0.4.0/) to verify their sexual specificity.

### 2.6. Validation of female-specific markers

To conﬁrm the authenticity of candidate sex-specific markers, we used gDNA samples of the gonad and podia tissues obtained from 10 female and 10 male re-sequenced individuals to perform the first round of screening by PCR amplification. To avoid the influence of experimental problems on amplification, we designed another pair of primers based on 18S rDNA sequence as a positive control (Supplementary Table S1) and performed PCR amplification using two sets of primers. Each PCR was carried out in a total volume of 20 μL, containing 10 μL 2 × Magic Green Taq SuperMix (ToloBio, China), 0.4 μL forward and reversed primers for the female-specific sequence, and 18S rDNA (10 μM), 2 μL DNA template (10-fold dilution) and 6.4 μL ddH_2_O as following: 95°C for 10 min; 30 cycles of 95°C for 15 s, 55°C for 20 s and 72°C for 1 min; and 72°C for 5 min. The PCR products were examined on 1% agarose gels and we sequenced the products of female-specific sequences using the Sanger approach (BGI, China). After initial verification, two validated sex-specific markers were further used to verify the genetic sex of another 10 female and 10 male *A. amurensis* by using gDNA from podia tissue.

We utilized blastn (v2.14.1)^55^ to locate these two sex-specific sequences (C2132450:490-2,028 and C4341577:1-1,155) on the published female genome.^56^ MUMmer (v4.0.0rc1)^57^ was used to align the reference genome of two sexes so that we could confirm the relationship between the different chromosomes of two genomes.

## 3. Results

### 3.1. Statistics of sequencing data and SNP calling

We obtained a total of ~398.43 Gb raw data from whole-genome re-sequencing of 40 female and 40 male *A. amurensis*, with a mean sequencing depth of ~10.1×. After quality control, approximately 383.91 Gb clean reads were mapped to the reference genome with an average mapping rate of 99.40% (from 98.86% to 99.54%) (Supplementary Table S2). The GATK pipeline identified a total of 3,7481,260 SNPs, of which 396,099 were retained after quality control and used for further genome-wide analysis.

### 3.2. Whole‑genome sequence association analysis

**Fig. 1.**
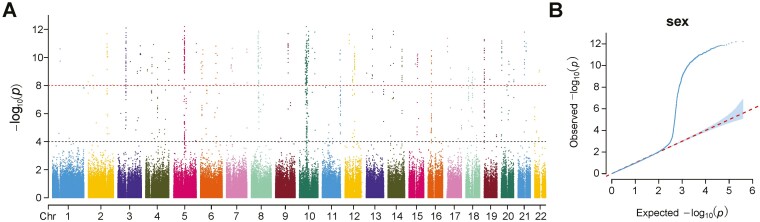
Manhattan and QQ plot of genome-wide sex-associated SNPs in *A. amurensis*. (A) Manhattan plot displaying GWAS results between male and female *A. amurensis*. The X-and Y-axis represents the physical location of all SNPs across chromosomes 1-22 and corresponding -log_10_ (*P*-value), respectively. The upper and lower dotted threshold lines represent ‘P-value = 1e-4’ and ‘P-value = 1e-8’, respectively. (B) QQ plot from SNP association analysis. The X-axis represents the expected observed -log_10_ (P-value) under the assumption that P-values follow a uniform distribution and the Y-axis represents the observed -log_10_ (*P*-value) from SNP association analysis. The blue region shows a 95% confidence interval under the null hypothesis of no association between the SNP and the sex.

The reference genome assembly was consistent with the karyotype analysis, indicating that *A. amurensis* has a diploid chromosome number of 44.^27,58^ For the GWAS of binary sex trait, a Manhattan map was created to show the distribution of sex-associated loci on the chromosomes, and a QQ-plot test was performed for quality control. A total of 525 significant sex-associated SNPs were identified with the threshold of *P *< 1e−8 (Fig. 1A). QQ plot showed that the SNPs started to separate from the uniform distribution rapidly when the *P*-value was < 1e−4, which indicated the occurrence of natural selection instead of random genetic drift (Fig. 1B).

### 3.3. Identification of sex-determining region

**Fig. 2.**
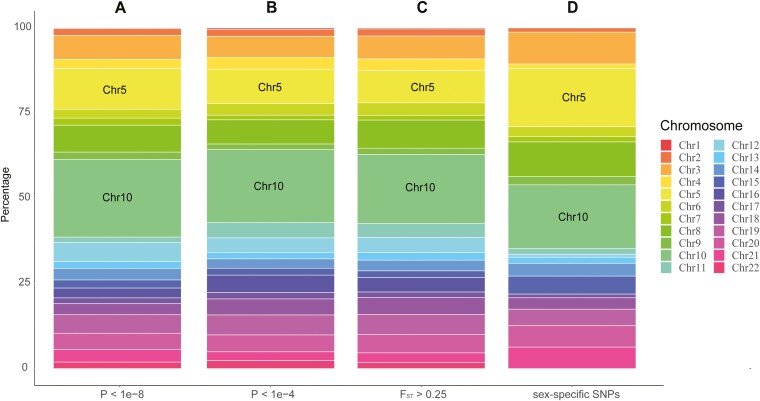
Chromosome distribution of the selected loci. The X-axis refers to the significant sex-associated SNPs (*P*-value* *< 1e−8) (A), the sex-associated SNPs (*P*-value* *< 1e−4) (B), the loci with F_ST_ values greater than 0.25 (C), and the sex-specific loci isolated from the custom Perl script (D). The Y-axis represents the percentages of the selected loci per unit chromosome length for each chromosome.

The results of GWAS indicated that the significant sex-associated SNPs (*P *< 1e−8) were mainly located on chromosome 10 (22.86%) and chromosome 5 (14.86%) (Fig. 2A). Consistent results were obtained by changing the threshold (*P *< 1e−4) (Fig. 2B). To provide more evidence for potential sex chromosomes, we calculated F_ST_ values of all identified SNPs. The loci whose F_ST_ values exceeded 0.25 indicated significant genetic differentiation. The chromosome-level statistics of F_ST_ values exceeding 0.25 supported that chromosome 10 (20.28%) and chromosome 5 (9.47%) were the main candidate chromosomes (Fig. 2C). In addition, 119 sex-specific loci were isolated by the custom Perl script, of which 118 loci were mostly heterozygotic in tested females and homozygotic in males, indicating that *A. amurensis* might have a ZZ/ZW sex-determination system. In addition, these loci were mainly located on chromosome 5 and chromosome 10 (Fig. 2D). We also found that an SNP on chromosome 10 was heterozygotic in all tested females and homozygotic in all males, which could be used to distinguish the two sexes.

**Fig. 3.**
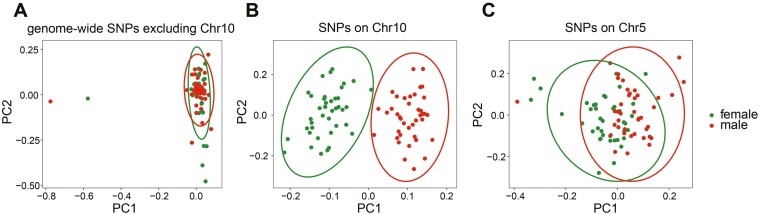
Principal component analysis of 80 individuals using genome-wide SNPs excluding SNPs on chromosome 10 (A), SNPs on chromosome 10 (B), and chromosome 5 (C).

Then, we conducted the PCA analysis for SNP sets on different chromosomes and genome-wide SNPs (except SNPs on chromosome 10). Only the SNPs located on chromosome 10 could clearly distinguish 80 individuals into females and males (Fig. 3 and Supplementary Fig. S1), which indicated that the genetic variations between the two sexes were mainly located on chromosome 10.

**Fig. 4.**
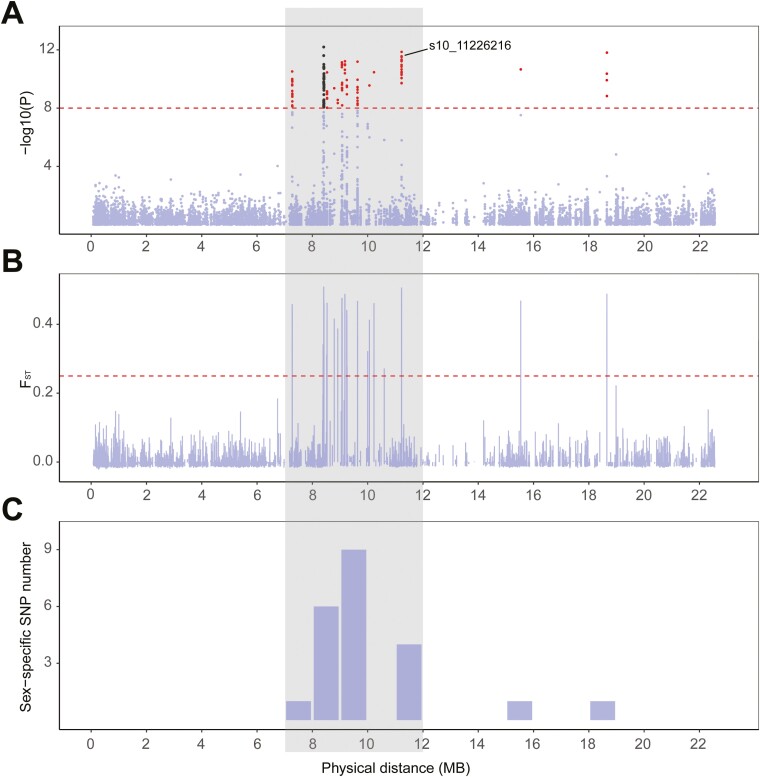
Identification of the candidate sex determination region on chromosome 10 of *A. amurensis*. (A) GWAS results on sex of *A. amurensis*. The X-and Y-axis represent the physical location of SNPs on chromosome 10 and corresponding -log_10_ (*P*-value), respectively. The dotted threshold line represents ‘P-value = 1e-8’. The black dots represent SNPs located on 3’UTR, upstream and downstream of Aam_011084, Aam_011085, and Aam_011086. (B) Distribution of F_ST_ values. The X-and Y-axis represents the physical location of SNPs on chromosome 10 and corresponding F_ST_ values, respectively. The red dotted threshold line represents ‘F_ST_ value = 0.25’. (C) Distribution of sex-specific loci isolated from the Perl script. The X-and Y-axis represents the sliding window of 1 Mb and corresponding numbers of sex-specific loci.

The Manhattan map indicated that the sex-associated SNPs of chromosome 10 (95.83%) were mainly located in the physical position from 7 Mb to 12 Mb. As shown in Fig. 4, this region showed higher F_ST_ values and sex-specific loci numbers than adjacent regions. Thus, the physical region on chromosome 10 (7-12 Mb) was considered to be the candidate sex-determining region. It is worth noting that 13-14 Mb region on chromosome 5 showed similar features to the candidate sex-determining region (Supplementary Fig. S2), though SNPs located on chromosome 5 could not distinguish sex (Fig. 3C).

### 3.4. Exploring candidate genes for sex determination

A total of 525 significantly sex-associated SNPs (*P* < 1e−8) were annotated by SnpEff software to predict their functional effects (Supplementary Table S3). In brief, 104 genes were associated with 497 variants, which were categorized into 1 high-impact effect, 8 moderate-impact effects, 4 low-impact effects, and 484 modifier-impact effects. The only high-impact effect was a stop_lost and splice region variant, which affected an uncharacterized gene (Aam_009174). In addition, 79 modifier-impact effect variants were located on 3ʹUTR, upstream and downstream of three uncharacterized genes (Aam_011084, Aam_011085, and Aam_011086) in the candidate sex-determining region, while their functions were uncertain by BLAST-based annotation. These uncharacterized genes need to be focused on in future functional research.

### 3.5. Development of sex-specific markers

**Fig. 5.**
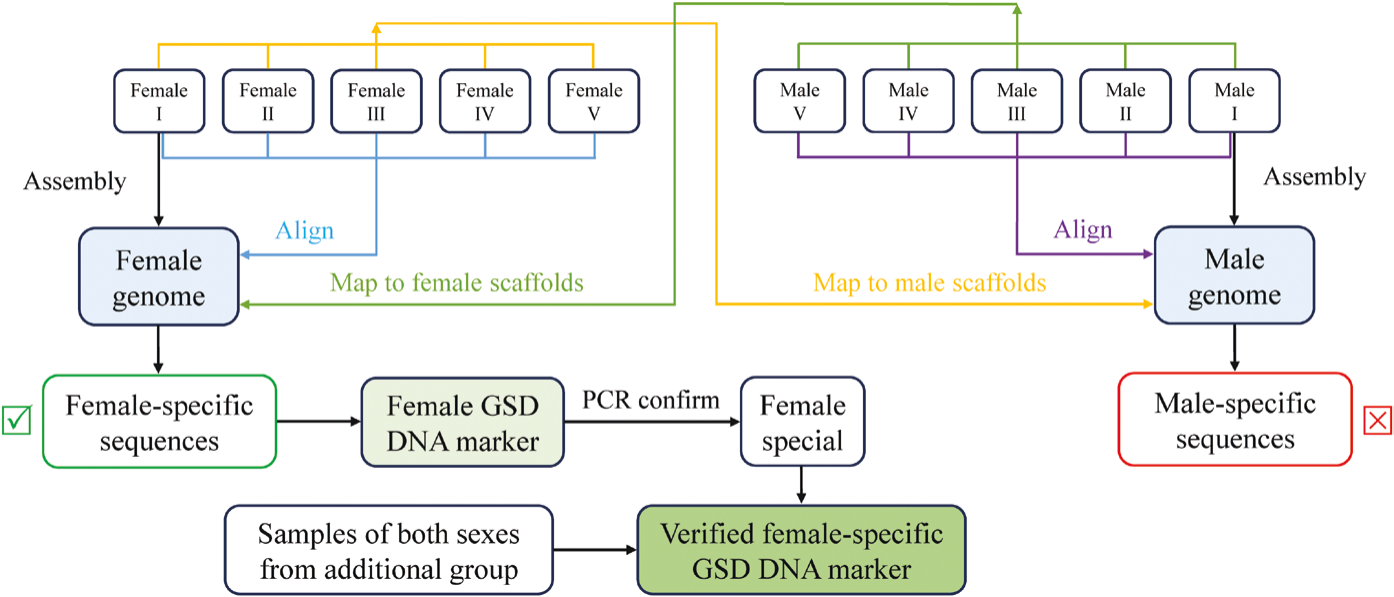
The general pipeline for screening the sex-specific molecular markers in *A. amurensis*.

The general pipeline for the development of *A. amurensis* sex-specific markers is shown in Fig. 5. Based on the re‑sequencing data of F04 and M03, the SOAPdenovo2 was applied for *de novo* assembly of two sexes. A total of 1,229,875 and 1,134,554 sequences were obtained with an N50 of 530 bp and 581 bp in female and male assembled genomes, respectively (Supplementary Table S4). After reads mapping with 5 female and 5 male libraries (Supplementary Table S5) and an artificial screen using pooled data from 30 males, we identified 277 female-specific sequences and no male-specific sequence only covered with corresponding sex sequencing reads (Supplementary Table S6 and Supplementary Fig. S3). In the process of artificial screening, the false positive sequences, sequences with a large number of ‘N’ bases, and repeats were removed. The identification of female-specific sequences further supported the ZZ/ZW sex determination system of *A. amurensis*. The fragments greater than 1000 bp in length were screened to design female-specific markers (Supplementary Table S1).

### 3.6. Validation of sex-specific markers

**Fig. 6.**
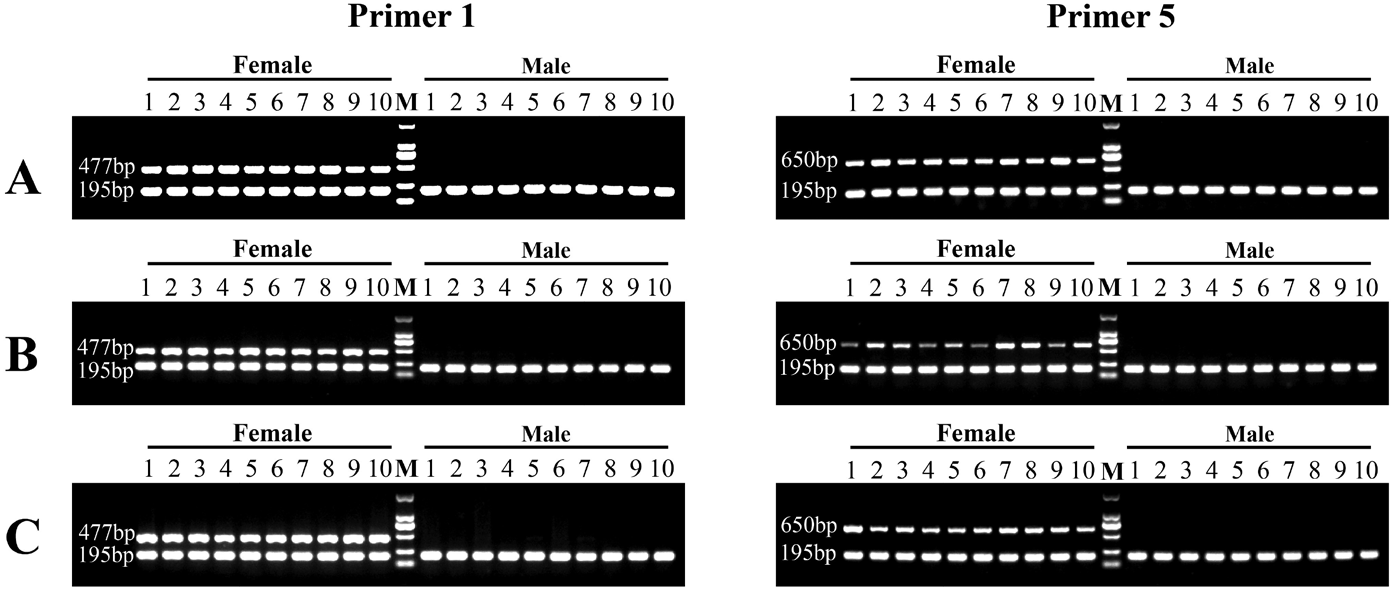
The validation of two pairs of sex-specific markers (Primer1 and Primer5) in two rounds of screening using agarose gel separation. The first round of screening used the gonad (A) and podia tissue (B) from 10 female and 10 male re-sequenced *A. amurensis*. The second round of screening used the podia tissue from another 10 females and 10 males (C). M represents DL 2000 DNA marker. The PCR amplification products using Primer1 and Primer5 are 477 bp and 650 bp, respectively. The PCR amplification product using Primer6 (positive control) is 195bp.

For the first round of verification, we used 20 re-sequenced *A. amurensis* (10 females and 10 males) to evaluate the reliability and usability of five pairs of designed primers. As a result, two pairs (Primer1 and Primer5) successfully identified the genetic sex with 100% accuracy. A specific amplification product was observed in female samples, while no target band was observed in male samples (Fig. 6A,B). To further confirm the practicability of these markers, they were tested in another 10 females and 10 males, which were sampled a year later in natural waters during sexual maturity of *A. amurensis* (Fig. 6C). The result indicated that the genetic sex of *A. amurensis* identified using these female-specific markers was consistent with their phenotypic sex. The PCR products amplified by Primer1 and Primer5 in females were sequenced using the Sanger method and confirmed that they were excellent sex-specific markers of *A. amurensis* (Supplementary Figs. S4 and S5).

In addition, we found one sequence (C2132450:490-2028) on the female genome^56^ which has two copies located at 12,831,722bp and 13,948,556bp on chromosome 11. In addition, chromosome 11 in the female genome corresponded to chromosome 10 in the male reference genome^27^ (Supplementary Fig. S6, Supplementary Table S7). As for the other sequence (C4341577:1-1155), we were unable to locate it on the female genome assembly.

## 4. Discussion

In this study, we re-sequenced 40 female and 40 male *A. amurensis* to explore the mechanism of sex determination, which led to the successful identification of two pairs of female-specific molecular markers that can distinguish the genetic sex of *A. amurensis*.

Our understanding of the mechanisms that define sex determination in echinoderms is very limited.^21^ Up to now, most studies have focused on various economically important aquaculture species, such as the sea cucumbers *A*. *japonicus*^21,22,59^ and *S. monotuberculatus*,^23^ as well as the sea urchins *M. nudus*,^24,25,60^*Strongylocentrotus intermedius*^61^ and *S. purpuratus*.^62^ Yet, related studies had not yet been applied to sea stars, brittle stars, and sea lilies. The genotypes of sex-specific SNPs suggested that the *A. japonicus* has an XX/XY sex-determination system,^21,22^ whereas in *S. monotuberculatus*, k-mer distribution, and sex-specific sequence length distribution inferred that it utilizes a male heterogametic sex determination mechanism.^23^ Karyotype analysis of the sea urchin *P. lividus* indicated that it likely has a heteromorphic chromosome sex mechanism of the XY type.^26^ While in another sea urchin, *M. nudus*, female-specific sequences and female heterozygous SNP loci suggested a ZW/ZZ sex determination system.^24,25^ In the present study, almost all sex-specific loci (118 out of 119) were heterozygotic in females and homozygotic in males on the whole genome. When screening sex-specific markers, we identified a total of 277 female-specific sequences but no male-specific sequences. Thus, based on our accumulative evidence, we suggest that *A. amurenis* owns a ZZ/ZW sex determination system.

For the northern Pacific seastar, *A. amurensis*, no representative sex chromosomes are observed following karyotype analysis.^58^ However, in the present study, we observed a broad distribution of sex-associated SNPs on different *A. amurensis* chromosomes. Consistent with our observation, a recent study also found an abundance of sex-associated SNP loci on almost every chromosome of *A. japonicus*, suggesting that the broad distribution of sex-related SNPs maybe a common phenomenon in echinoderms.^63^ To the best of our knowledge, this is the ﬁrst study to explore the sex-specific variations in starfish. Further research on more echinoderm species should be conducted for confirmation.

Our published chromosome-level reference genome of *A. amurensis*^27^ provided the basis for further in-depth analysis of its mechanism of sex determination. Combined evidence based on the distribution characteristics of sex-associated SNPs and sex-specific loci, the selection signatures of F_ST_ and PCA analysis, all supported that a 7-12 Mb region on chromosome 10 was the candidate sex-determination region in *A. amurensis*. This physical region contained abundant sex-associated SNPs and loci with high F_ST_ values, which is consistent with observations in previous studies, that sites associated with high F_ST_ values tend to coincide with the sex-determining locus identified by GWAS analysis.^21,32,48,64,65^ Due to the occurrence of missing data and low coverage depth, several heterozygotic SNPs might be miscalled as homozygotic SNPs.^48^ Therefore, we set a minimum of 25 heterozygotes in one sex and no heterozygote in the corresponding sex to find sex-specific SNPs. In the candidate sex-determining region, a total of 21 sex-specific SNPs were identified, including s10_11226216, which was completely heterozygotic in females and homozygotic in males. Although there are more sex-specific SNPs on chromosome 5 (25) compared to chromosome 10, PCA analysis indicated that SNPs on chromosome 10 could more accurately distinguish between males and females. Thus, the 7-12 Mb region of chromosome 10 is a more important candidate sex-linked region in *A. amurensis*, which should be targeted in future sex-associated studies.

To obtain the list of candidate sex-determination genes in *A. amurensis*, we conducted functional annotation of the sex-associated SNPs, leading to the identification of three uncharacterized genes, which might play potential roles in sex determination/differentiation. Recently, various candidate genes linked with sex determination have been reported in several aquatic animals,^24,29,31,48,66,67^ suggesting no universal master sex determination gene in aquatic species. Therefore, the candidate genes identified in our present study will provide targets for future functional research on sex determination in starfish.

To our knowledge, several non-lethal sex identification methods have been described other than molecular markers in echinoderms, which are mainly based on inducing spawning or gonad collection with microscopy, as well as observation of secondary sexual characteristics (e.g. genital papillae).^68,69^ However, these methods are limited to some inevitable disadvantages, such as being seasonally dependent, occasionally resulting in mortality, and not being accurate enough. Comparatively speaking, molecular markers like those developed in this study only need a small amount of tube foot for DNA isolation and could distinguish the genetic sex of *A. amurensis* with 100% accuracy. In addition, this approach is noninvasive, reliable, and has been applied for sex identification in several echinoderm species.^22,23,25,34^ Considering that extensive tissue-level sexual dimorphism might play a role in the aggregation and spawning of starfish,^70^ our developed sex-specific molecular markers could also contribute to future studies into the function of sex-specific pheromones in *A. amurensis* and provide novel research perspectives for starfish aggregation and outbreaks.

## 5. Conclusion

Sex determination systems are diverse in echinoderms, however, our understanding is still very limited in this research field, especially for Asteroidea species. In the present study, we re-sequenced 40 female and 40 male *A. amurensis* to explore the mechanism of sex determination and develop sex-specific molecular markers. Combined with the distribution characteristics of sex-associated SNPs and sex-specific loci, the selection signatures of F_ST,_ and PCA analysis, the physical region of 7-12 Mb on chromosome 10 was identified as the candidate sex determination region in *A. amurensis*. The existence of female-specific sequences and the genotypes of sex-specific loci indicated that *A. amurensis* might utilize a ZZ/ZW sex-determination system. What’s more, two pairs of female-specific primers were identified by the NGS method, which was proven to successfully distinguish the genetic sex of *A. amurensis*. As the first study on sex determination in Asteroidea, our results provide novel insights into diverse sex determination systems in echinoderms and contribute to future sex-related eco-physiological research in *A. amurensis*.

## Supplementary Material

dsaf003_suppl_Supplementary_Figure_S1

dsaf003_suppl_Supplementary_Figure_S2

dsaf003_suppl_Supplementary_Figure_S3

dsaf003_suppl_Supplementary_Figure_S4

dsaf003_suppl_Supplementary_Figure_S5

dsaf003_suppl_Supplementary_Figure_S6

dsaf003_suppl_Supplementary_Materials

dsaf003_suppl_Supplementary_Tables_S1

dsaf003_suppl_Supplementary_Tables_S2

dsaf003_suppl_Supplementary_Tables_S3

dsaf003_suppl_Supplementary_Tables_S4

dsaf003_suppl_Supplementary_Tables_S5

dsaf003_suppl_Supplementary_Tables_S6

dsaf003_suppl_Supplementary_Tables_S7

## Data Availability

The raw re-sequencing data has been deposited in the NCBI Sequence Read Archive (SRA) under BioProject Accession No. PRJNA1120217. Other data supporting the findings of this study are available in the main manuscript and additional supporting files.
