# Supplementary figures and images for: Identification of a candidate sex determination region and sex-specific molecular markers based on whole-genome re‑sequencing in the sea star Asterias amurensis
Source: DNA Res. 2025 Jan 10;32(1):dsaf003. doi: 10.1093/dnares/dsaf003 (PMC11757944; doi:10.1093/dnares/dsaf003)

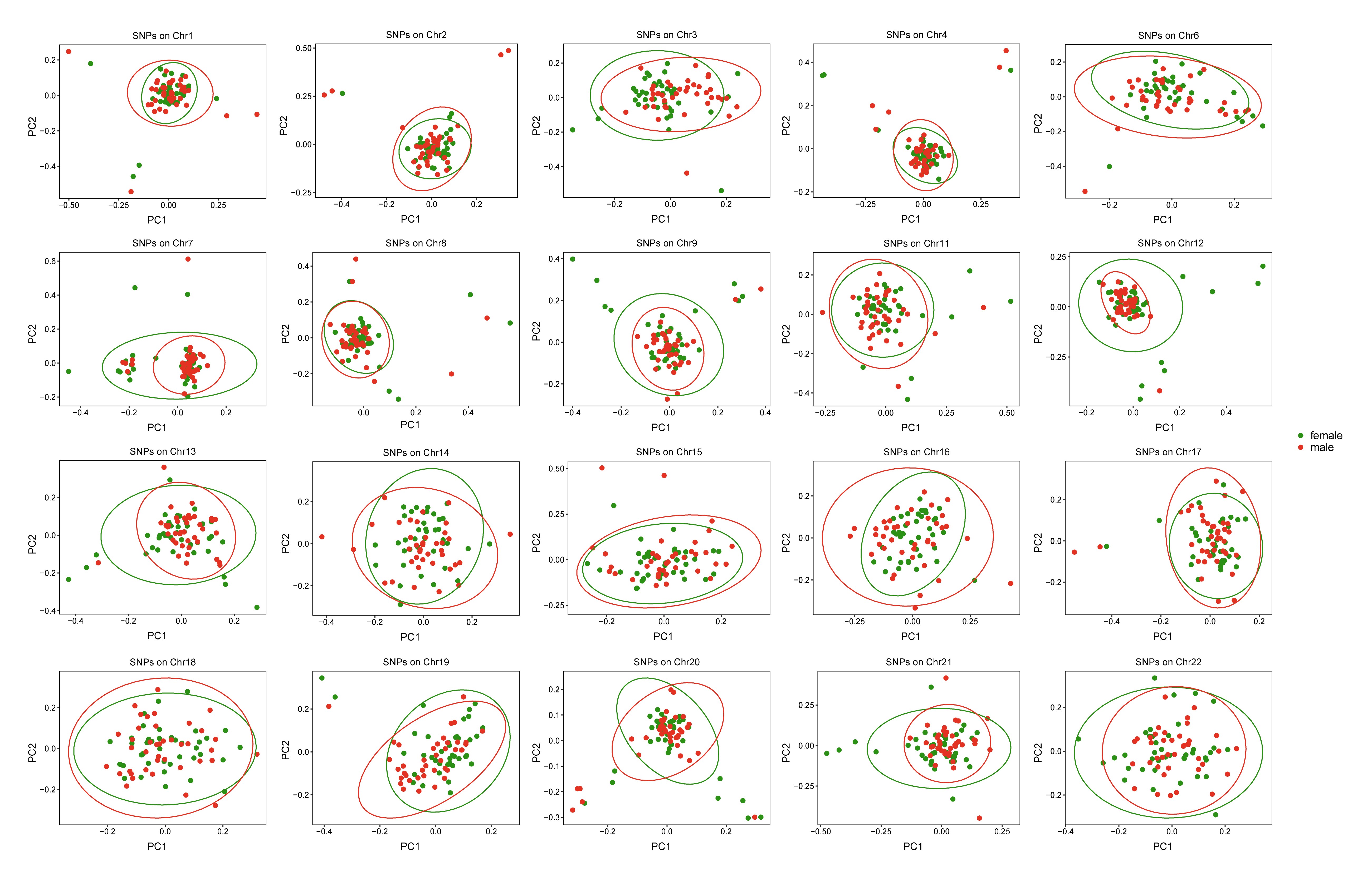

Supplement: dsaf003_suppl_Supplementary_Figure_S1 [file dsaf003_suppl_supplementary_figure_s1.jpeg]

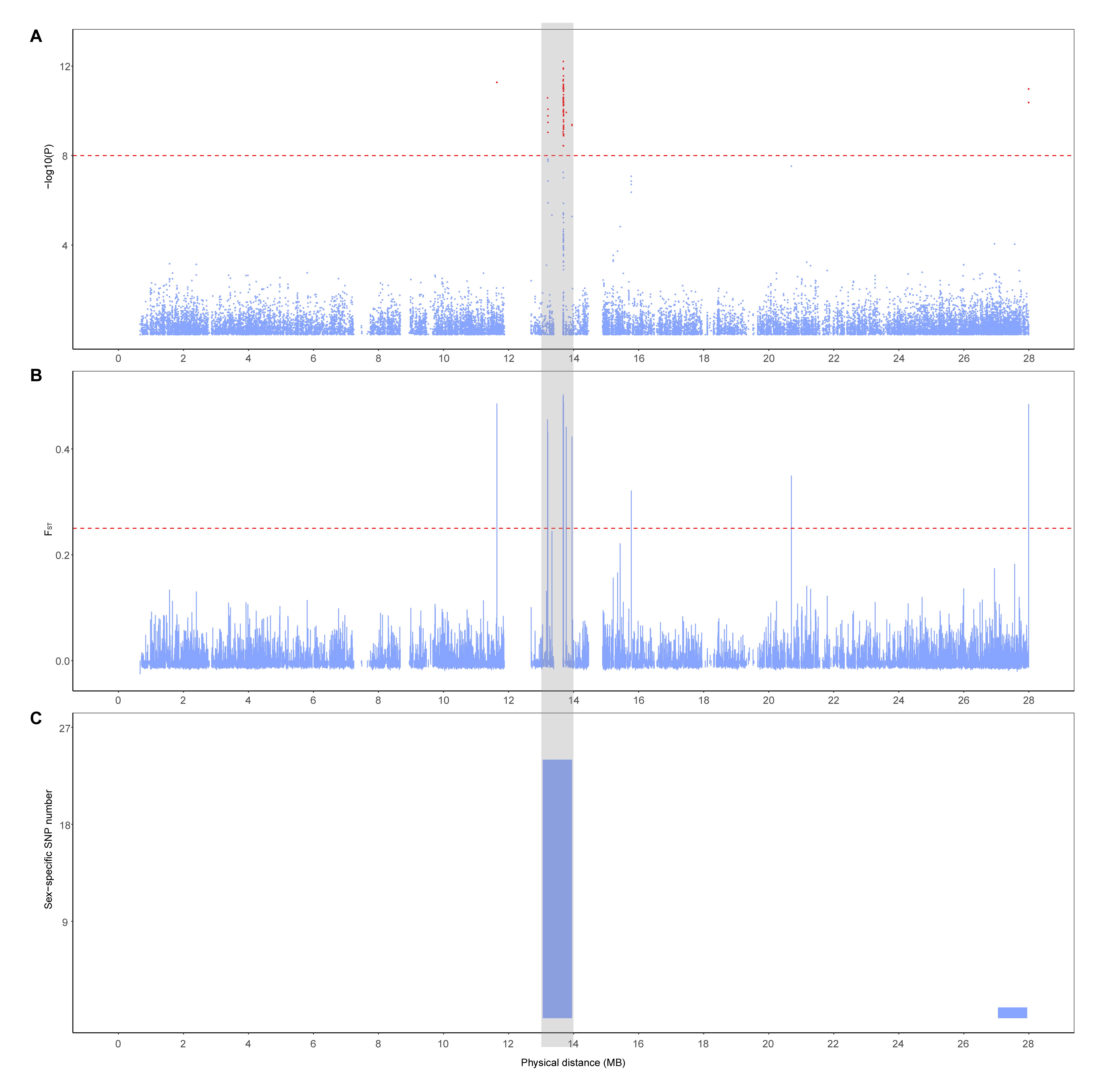

Supplement: dsaf003_suppl_Supplementary_Figure_S2 [file dsaf003_suppl_supplementary_figure_s2.jpeg]

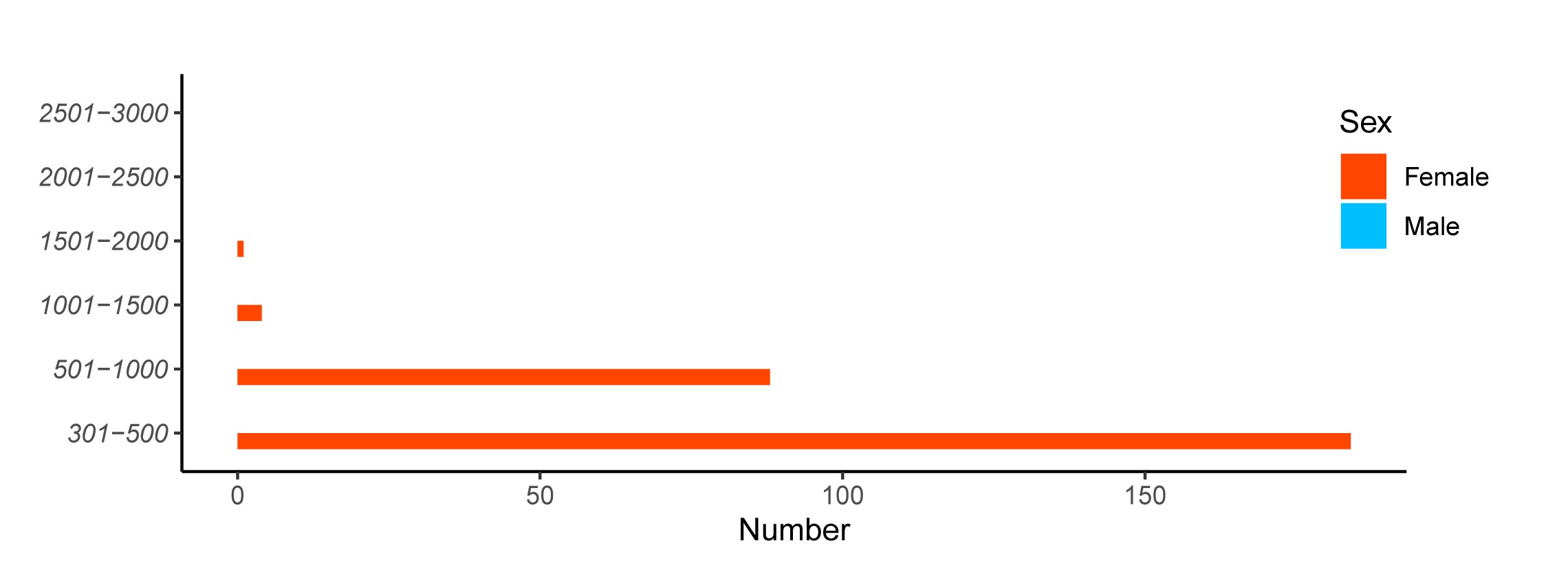

Supplement: dsaf003_suppl_Supplementary_Figure_S3 [file dsaf003_suppl_supplementary_figure_s3.jpeg]

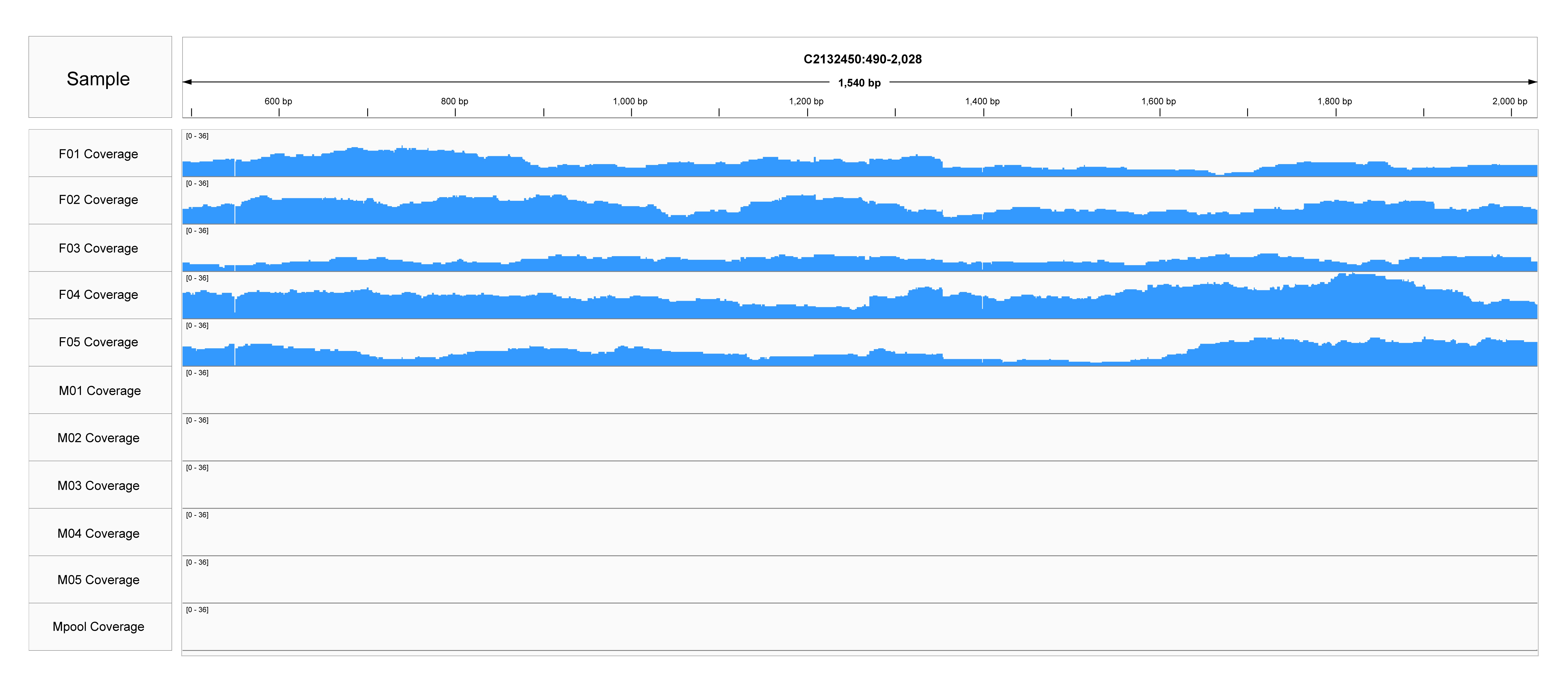

Supplement: dsaf003_suppl_Supplementary_Figure_S4 [file dsaf003_suppl_supplementary_figure_s4.jpeg]

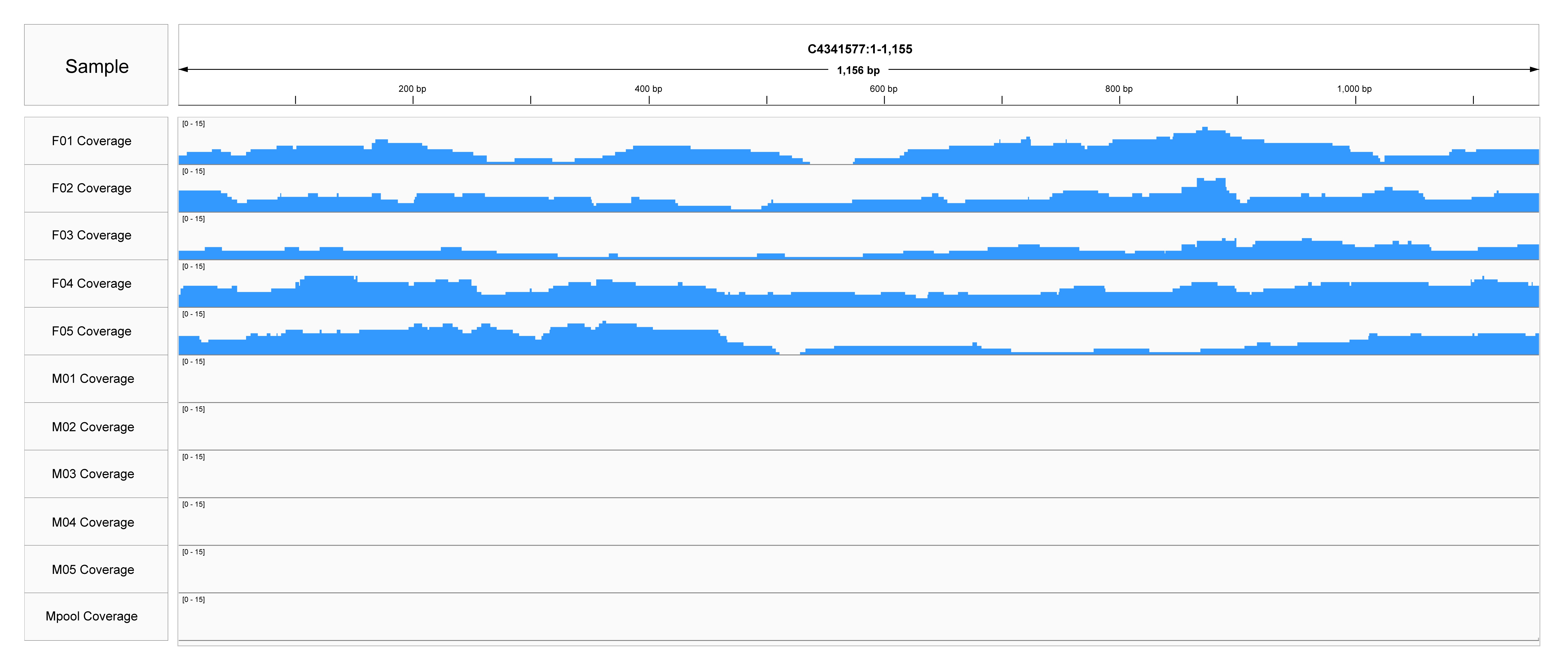

Supplement: dsaf003_suppl_Supplementary_Figure_S5 [file dsaf003_suppl_supplementary_figure_s5.jpeg]

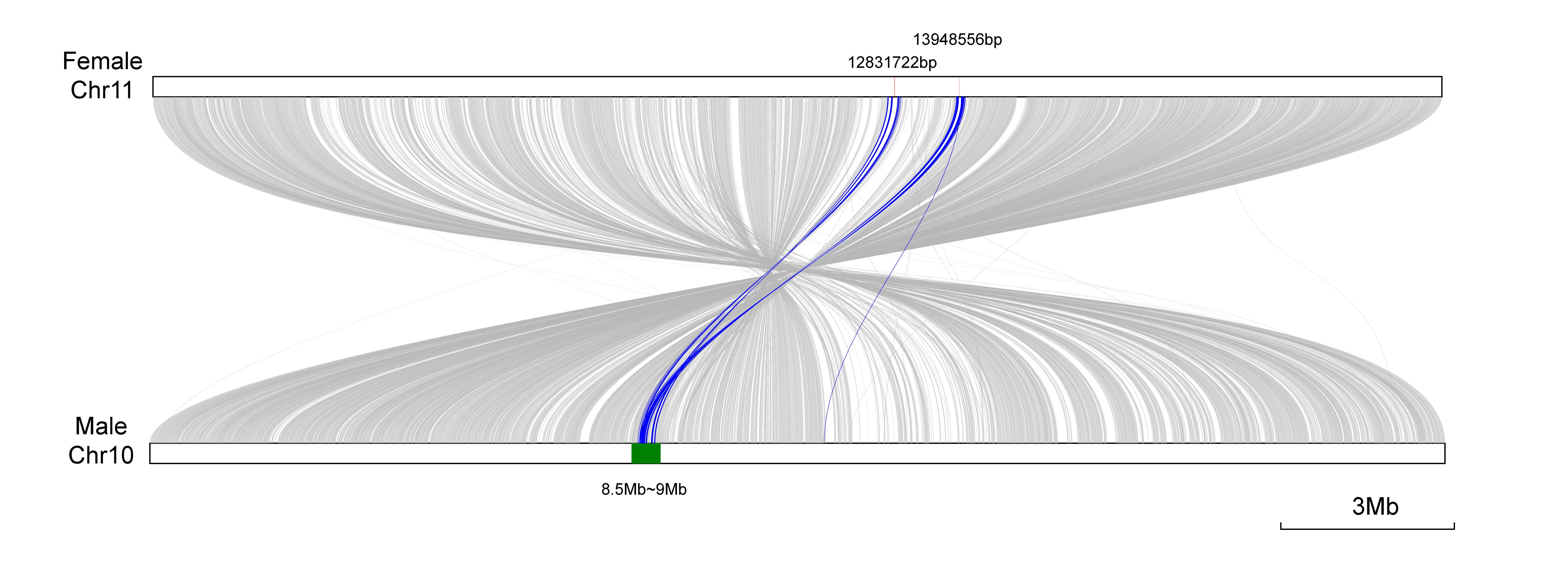

Supplement: dsaf003_suppl_Supplementary_Figure_S6 [file dsaf003_suppl_supplementary_figure_s6.jpeg]
