# Supplementary material for: Identification of a candidate sex determination region and sex-specific molecular markers based on whole-genome re‑sequencing in the sea star Asterias amurensis
Source: DNA Res. 2025 Jan 10;32(1):dsaf003. doi: 10.1093/dnares/dsaf003 (PMC11757944; doi:10.1093/dnares/dsaf003)
Supplement: dsaf003_suppl_Supplementary_Materials [file dsaf003_suppl_supplementary_materials.docx]

**Supplementary Figure S1.** Principal component analysis of 80 individuals using SNPs on different chromosomes.

**Supplementary Figure S2.** Identification of the candidate sex determination region on chromosome 5. (A) GWAS results on sex of *A. amurensis*. The X-and Y-axis represent the physical location of SNPs on chromosome 5 and corresponding -log_10_ (P-value), respectively. The red dotted threshold line represents “P-value = 1e-8”. (B) Distribution of F_ST_ values. The X-and Y-axis represent the physical location of SNPs on chromosome 5 and corresponding F_ST_ values, respectively. The red dotted threshold line represents “F_ST_ value = 0.25”. (C) Distribution of sex-specific loci isolated from the Perl script. The X-and Y-axis represent the sliding window of 1 Mb and corresponding numbers of sex-specific loci.

**Supplementary Figure S3.** The length and number distribution of female-specific and male-specific sequences after artificial screening.

**Supplementary Figure S4.** Comparative alignments of the sequencing reads from both sexes on C2132450:490-2,028 (location of Primer1) visualized by IGV. Mpool represents the pooled data of 30 male *A. amurensis* (ranging from individual M01 to individual M30).

**Supplementary Figure S5.** Comparative alignments of the sequencing reads from both sexes on C4341577:1-1,155 (location of Primer5) visualized by IGV.

**Supplementary Figure S6.** The alignment between chromosome 11 in female reference genome and chromosome 10 in male reference genome. The red lines represent positions where female-specific sequence (C2132450:490-2,028) is located on the female genome.
