# Supplementary material for: Identification of a candidate sex determination region and sex-specific molecular markers based on whole-genome re‑sequencing in the sea star Asterias amurensis
Source: DNA Res. 2025 Jan 10;32(1):dsaf003. doi: 10.1093/dnares/dsaf003 (PMC11757944; doi:10.1093/dnares/dsaf003)
Supplement: dsaf003_suppl_Supplementary_Tables_S1 [file dsaf003_suppl_supplementary_tables_s1.docx]

Supplementary Table S1.

Five pairs of candidate primers for verification of *A. amurensis* female-specific sequences (Primer1-5) and one pair of primers for positive control (Primer6).

| Sequence ID | Primer ID | Primer sequence | Product length |
| --- | --- | --- | --- |
| C2132450:490-2028 | Primer1_F | GTTCAAAACATGCCCGAAGT | 477 bp |
|  | Primer1_R | GGCACCAATGACCTTTCTGT |  |
| scaffold60002:91-1177 | Primer2_F | TCGGGCATTAGATCACCTTC | 450 bp |
|  | Primer2_R | TCTGGGGTTTTTCTCTCCCT |  |
| scaffold36247:140-1399 | Primer3_F | AGGGAAAAAGTTGCCCCTTA | 967 bp |
|  | Primer3_R | GCACCTCGATCTTTATCCCA |  |
| C4324631:160-1196 | Primer4_F | GTGGGGAGAGGCAGATTGTA | 690 bp |
|  | Primer4_R | CCCCCGATCACTGTGTAAGT |  |
| C4341577:1-1155 | Primer5_F | CCGGTTCGATTGAATGACTT | 650 bp |
|  | Primer5_R | CCCACCAGAGGATCTTTTCA |  |
| 18S_rRNA | Primer6_F | GCTCCGTTGGTGAACTCTG | 195bp |
|  | Primer6_R | GCTGCCTTCCTTGGATGTG |  |
