# Supplementary material for: Identification of a candidate sex determination region and sex-specific molecular markers based on whole-genome re‑sequencing in the sea star Asterias amurensis
Source: DNA Res. 2025 Jan 10;32(1):dsaf003. doi: 10.1093/dnares/dsaf003 (PMC11757944; doi:10.1093/dnares/dsaf003)
Supplement: dsaf003_suppl_Supplementary_Tables_S4 [file dsaf003_suppl_supplementary_tables_s4.docx]

Supplementary Table S4.

The statistical results of assembled genomes from female and male *A. amurensis*.

| Type | Female (F04) | Male (M03) |
| --- | --- | --- |
| Total size (bp) | 461,493,127 | 462,634,400 |
| Sequence number | 1,229,875 | 1,134,554 |
| Average length (bp) | 375 | 407 |
| Max_Length (bp) | 7,411 | 16,503 |
| Min_Length (bp) | 100 | 100 |
| seqs>=10kb number | 0 | 1 |
| seqs>=2kb number | 9,767 | 11,572 |
| N50 (bp) | 530 | 581 |
| GC Content (%) | 38.04 | 38.32 |
