# Supplementary material for: Identification of a candidate sex determination region and sex-specific molecular markers based on whole-genome re‑sequencing in the sea star Asterias amurensis
Source: DNA Res. 2025 Jan 10;32(1):dsaf003. doi: 10.1093/dnares/dsaf003 (PMC11757944; doi:10.1093/dnares/dsaf003)
Supplement: dsaf003_suppl_Supplementary_Tables_S5 [file dsaf003_suppl_supplementary_tables_s5.docx]

Supplementary Table S5.

The statistics of sequence alignment from 5 female and 5 male *A. amurensis*.

| Sample | Total reads | Reference (Female) | | | | Reference (Female) | | | |
| --- | --- | --- | --- | --- | --- | --- | --- | --- | --- |
|  |  | Match reads | Match Reads Radio(%) | PE Mapped Reads | PE Mapped Radio (%) | Match reads | Match Reads Radio(%) | PE Mapped Reads | PE Mapped  Reads (%) |
| F01 | 32,942,866 | 32,337,348 | 98.16 | 18,006,428 | 54.66 | 32,218,595 | 97.80 | 18,751,088 | 56.92 |
| F02 | 30,712,824 | 30,141,472 | 98.14 | 17,278,644 | 56.26 | 30,028,747 | 97.77 | 18,006,240 | 58.63 |
| F03 | 23,938,356 | 23,472,690 | 98.05 | 12,983,000 | 54.24 | 23,383,016 | 97.68 | 13,600,906 | 56.82 |
| F04 | 29,362,526 | 28,968,519 | 98.66 | 16,212,630 | 55.22 | 28,639,092 | 97.54 | 15,904,416 | 54.17 |
| F05 | 30,888,620 | 30,323,173 | 98.17 | 16,235,832 | 52.56 | 30,203,479 | 97.78 | 16,955,908 | 54.89 |
| M01 | 31,370,646 | 30,622,406 | 97.61 | 16,711,726 | 53.27 | 30,519,922 | 97.29 | 17,445,238 | 55.61 |
| M02 | 28,263,224 | 27,669,022 | 97.90 | 15,752,372 | 55.73 | 27,585,152 | 97.60 | 16,464,820 | 58.26 |
| M03 | 33,619,308 | 32,912,324 | 97.90 | 18,996,248 | 56.50 | 33,067,014 | 98.36 | 21,073,030 | 62.68 |
| M04 | 27,885,370 | 27,294,105 | 97.88 | 15,320,964 | 54.94 | 27,212,243 | 97.59 | 15,992,144 | 57.35 |
| M05 | 28,657,876 | 28,050,400 | 97.88 | 15,711,022 | 54.82 | 27,967,639 | 97.59 | 16,413,354 | 57.27 |
