# Supplementary material for: Identification of a candidate sex determination region and sex-specific molecular markers based on whole-genome re‑sequencing in the sea star Asterias amurensis
Source: DNA Res. 2025 Jan 10;32(1):dsaf003. doi: 10.1093/dnares/dsaf003 (PMC11757944; doi:10.1093/dnares/dsaf003)
Supplement: dsaf003_suppl_Supplementary_Tables_S6 [file dsaf003_suppl_supplementary_tables_s6.docx]

Supplementary Table S6.

The information of sex-specific sequences after screening.

| Sequence type | Sequence number | Total length (bp) | Max length (bp) | Min length (bp) | Average length (bp) |
| --- | --- | --- | --- | --- | --- |
| Female-specific | 277 | 133,078 | 1,539 | 300 | 480 |
| Male-specific | 0 | 0 | 0 | 0 | 0 |
