# Supplementary material for: Identification of a candidate sex determination region and sex-specific molecular markers based on whole-genome re‑sequencing in the sea star Asterias amurensis
Source: DNA Res. 2025 Jan 10;32(1):dsaf003. doi: 10.1093/dnares/dsaf003 (PMC11757944; doi:10.1093/dnares/dsaf003)
Supplement: dsaf003_suppl_Supplementary_Tables_S7 [file dsaf003_suppl_supplementary_tables_s7.docx]

Supplementary Table S7.

The correspondence of chromosome numbers in the male and female refence genome.

| **Male reference genome** | **Female reference genome** |
| --- | --- |
| chromosome 1 | chromosome 1 |
| chromosome 2 | chromosome 2 |
| chromosome 3 | chromosome 5 |
| chromosome 4 | chromosome 4 |
| chromosome 5 | chromosome 3 |
| chromosome 6 | chromosome 6 |
| chromosome 7 | chromosome 12 |
| chromosome 8 | chromosome 7 |
| chromosome 9 | chromosome 13 |
| chromosome 10 | chromosome 11 |
| chromosome 11 | chromosome 8 |
| chromosome 12 | chromosome 16 |
| chromosome 13 | chromosome 14 |
| chromosome 14 | chromosome 15 |
| chromosome 15 | chromosome 17 |
| chromosome 16 | chromosome 18 |
| chromosome 17 | chromosome 9 |
| chromosome 18 | chromosome 10 |
| chromosome 19 | chromosome 19 |
| chromosome 20 | chromosome 21 |
| chromosome 21 | chromosome 20 |
| chromosome 22 | chromosome 22 |
